# Conceptualizing problems with symptoms, function, health behavior, health-seeking skills, and financial strain in breast cancer survivors using hierarchical clustering

**DOI:** 10.1007/s11764-021-01068-w

**Published:** 2021-06-09

**Authors:** Xiangyu Liu, Yongyi Chen, Andy SK Cheng, Yingchun Zeng, Shahid Ullah, Michael Feuerstein

**Affiliations:** 1 Department of Health Service Center, The Affiliated Cancer Hospital of Xiangya School of Medicine, Central South University/Hunan Cancer Hospital, Changsha, China; 2 Department of Institute Office, The Affiliated Cancer Hospital of Xiangya School of Medicine, Central South University/Hunan Cancer Hospital, Changsha, China; 3 Department of Rehabilitation Sciences, The Hong Kong Polytechnic University, Hong Kong, China; 4 College of Medicine and Public Health, Flinders University, Adelaide, Australia; 5 Professor (Retired) Uniformed Services University, Bethesda, MD USA

**Keywords:** Breast cancer survivors, Hierarchical cluster analysis, Symptom burden, Functional limitations, Economic burden, Health-seeking skills

## Abstract

**Purpose:**

Determine whether a diverse set of problems experienced by breast cancer survivors (BCS) following curative treatment can be formulated into a reduced number of clusters, potentially simplifying the conceptualization of these problems.

**Method:**

Female BCS were recruited from four cancer hospitals in China. The Chinese translation of the Cancer Survivor Profile (CSPro) was used to measure 18 common problem areas, as supported by epidemiological and phenomenological research. The Functional Assessment of Cancer Therapy–Breast (FACT-B) was used to measure quality of life, as a validation of any observed groupings. Hierarchical clustering using multiple distance criteria and aggregation methods to detect patterns of problems was used.

**Results:**

A total of 1008 BCS (mean 46.51 years old) living in both urban and rural areas were investigated. Hierarchical cluster analysis identified two major clusters of problems. One set was classified as “functional limitations,” while the other cluster was labeled “multi-problems.” Those who fell into the multi-problem cluster experienced poorer quality of life.

**Conclusion:**

Eighteen non-medical problems were broken down into two major clusters: (1) limitations in higher level functions required of daily life and (2) limitations in health care–seeking skills, problems with certain symptoms, unhealthy behaviors, and financial problems related to cancer. The breakdown of problem areas into these two clusters may help identify common mechanisms.

**Implications for Cancer Survivors:**

In the future, the search for common clusters and the mechanisms for the many problems that breast cancer survivors and other cancer survivors can experience following primary treatment may improve how we help manage these problems in the future.

## Introduction

Over the past two decades, many patient-reported assessment tools have been developed to identify common problems experienced in cancer survivors following curative treatment [1–3]. These approaches have allowed for improved identification and development of corresponding interventions across many different problem areas [2, 3]. One such patient-reported tool is the Cancer Survivor Profile (CSPro) [4]. The CSPro was developed to detect a multidimensional range of symptoms, function-related challenges, lifestyle behaviors, financial strain, and difficulty with skills helpful in obtaining quality health care  for in breast cancer survivors (BCS) following primary oncology treatment and beyond.

The original goal of the CSPro was to provide a practical, valid, reliable, and relatively rapid assessment tool for the valid detection of a wide range of problem areas of breast cancer survivors. The selection of problem areas was based on a comprehensive review of both the epidemiological and qualitative literature [4, 5], resulting in identification of 18 distinct problems. Corresponding measures were carefully selected and found to have sound measurement features in BCS. Factor analysis and confirmatory factor analysis indicated the resulting CSPro measured problems across the following domains: health care–seeking skills (healthcare competence, health information, patient-provider communication. health information, information acquisition), symptoms (fear of cancer recurrence, poor body image, pain, fatigue, depressive symptoms, anxiety), function (cognitive, social, sleep, work, and sexual function), health behavior (low levels of physical activity and unhealthy diet), and economic strain [4]. The CSPro has been translated into Chinese and this version has also been rigorously validated [6].

The CSPro was comprehensive by design. It was intended for scales measuring different problems to be relatively independent of each other and facilitate identification of specific challenges across several many problem areas. While this may serve a helpful function (i.e., intervention targeting), the comprehensiveness of 18 distinct problem areas presents its own challenges. For example, when using the CSPro as a tool in a clinical setting, BCS reported difficulty with the number of individual areas, noting that it was confusing/overwhelming to pay attention to all these problems simultaneously [7]. Similarly, this level of comprehensiveness may have the unintended consequence of limiting investment of finite clinical and research resources (i.e., forcing reduced investment in time across 18 domains vs a greater focus on a select few). Because of these reasons, it was thought that reducing this diverse set of problems into more manageable groupings may be easier to understand and implement.  serve a helpful function. Specifically, this type of integration optimization may provide a simpler way to conceptualize and more efficiently and effectively manage these multiple problems. It may also help identify common underlying mechanisms in the future. The current study sought to achieve this simplification through cluster analysis.

The use of cluster analysis in “symptom” science [8] has generated an improved understanding of potential mechanisms that underlie many symptoms experienced by BCS. The use of clustering techniques to better understand how several individual problem areas (i.e., not limited to symptoms) might be related or nested into a few clusters [14] may also help identify common mechanisms of a multidimensional array of problems. It was reasoned that by following a similar methodology, a diverse set of problems in addition to symptoms, as measured by a tool such as the CSPro, may be reduced into more manageable groupings.

During the original development of the CSPro, it was observed that, despite independence of scales, there was some shared variance across problem areas [4]. Therefore, it was assumed that perhaps a more parsimonious set of problem areas might empirically emerge and assist in identifying common underlying pathways in the future. This effort might in turn provide a more efficient way to classify and manage the diverse set of the problems that can be observed in BCS.

## Methods

### Study design

This study was cross-sectional using random multicenter sampling. The study also investigated the relationship of any observed clusters to a standard measure of quality of life (QoL). This study followed the STROBE guidelines for the reporting cross-sectional studies [9]. Chinese-speaking adult patients diagnosed with breast cancer and completed curative treatment for breast cancer between February and October of 2020 were eligible to participate. The Hunan Cancer Hospital Breast Cancer Center institutional review board approved this study.

### Study population/recruitment

The inclusion criteria were (a) female, (b) diagnosed with breast cancer in stages I to III, (c) who completed primary therapy (surgery, chemotherapy, and/or radiation) within 2 years, (d) aged 18 or over, and (e) possessed an ability to understand all questions. A total of 1031 patients who met the study criteria and were randomly recruited across four hospitals. 1008 patients agreed to participate in this study and completed all surveys (completion rate = 97.8%). All survey measures were completed via telephone consultation with an oncology nurse. Clinical data were obtained through medical records. The 23 cases without complete data were deleted from all analyses. Analysis of the cases was investigated for differences in age, education, years from treatment, stage of cancer, and type of treatments with the final sample used. No differences were observed. The 1008 patients who did complete all measures were from four different Cancer Hospitals: Hunan Cancer Hospital/the Affiliated Cancer Hospital of Xiangya School of Medicine, Central South University (n = 550), Jiangxi Cancer Hospital (n = 110), Guangxi Cancer Hospital (n = 150), and Henan Cancer Hospital (n = 198).

### Measures

#### Sociodemographic and clinical characteristics

The full survey included measures of age, education, marital status, pregnancy history, work status, type of work, residence, and income and the CSPro questions. Clinical variables included years since diagnosis, treatment methods, pathological stage, and family history of cancer and obtained from medical records.

#### Multidimensional problems: the Cancer Survivor Profile

The Chinese translation of the Cancer Survivor Profile for breast cancer (CSPro) was used [6]. As with the English version, the survey includes seventy-one specific questions that measure multiple problem areas, including fear of recurrence, body image, pain, fatigue, depressive symptoms, anxiety, cognitive function, social function, sleep, work function, limitations in sexual function, physical inactivity, unhealthy diet, financial strain, and limited health care–seeking skills (i.e., healthcare competence, patient-provider communication, health information, and health information acquisition).

Using empirically based factor analysis, the scales formed five broader domains: symptom burden, functional limitations, health behavior, financial strain, and health care–seeking skills. The culturally sensitive Chinese translation was rigorously tested and possessed high levels of reliability (Cronbach’s α coefficients range − 0.87~0.92) and content validity [6]. The Chinese version observed that confirmatory factor analysis supported the original measurement models describing problem areas that were consistent with the original English version: symptom burden (CFI = 0.949, RMSEA = 0.055), functional limitations (CFI = 0.925, RMSEA = 0.080), health behavior (CFI = 0.999, RMSEA = 0.015), financial strain (CFI = 0.999, RMSEA = 0.014), and health care–seeking skills (CFI = 0.964, RMSEA = 0.059). The test-retest reliability for the Chinese version was between 0.80 and 0.92 and internal consistency ranged from 0.65 to 0.95 [6]. Calculation of the total score for each problem area was simply the addition of the raw scores of each item. Higher scores represented greater levels of the problem.

#### Quality of life–breast

The Chinese version of the Functional Assessment of Cancer Therapy–Breast (FACT-B) [10–12] was used as a gold standard measure of QoL. The FACT-B measures elements of quality of life in cancer patients with a specific module for breast cancer patients. The current study utilized all four FACT subscales (physiological status, social/family status, emotional status, functional status) and the additional breast cancer–specific FACT subscale B (nine items). The higher the total score, the greater the quality of life. The Chinese version of the FACT-B has acceptable reliability and validity and is applicable to many clinical periods in patients with breast cancer [12]. Cronbach’s α = 0.82. The total score was used in the current investigation.

### Statistical analysis

Statistical analyses were performed using R statistical software, version 4.0.4 [13]. Patient’s demographic and clinical characteristics were expressed as median and intra-quartile range (IQR) for continuously skewed data and proportions presented as percentages of the respective denominator. Mann-Whitney U-test and standard Chi-square tests for association with continuity correction were used to explore differences in patient’s characteristics between cluster 1 and cluster 2. Median and IQR for patient problems were calculated and Mann-Whitney U-tests used to explore the specific differences in problem areas between the two clusters.

Primary analyses were completed using hierarchical clustering with different distance measures and aggregation methods to identify clusters of problems experienced by BCS based on the five domains and the eighteen problem areas. Differences in the total score of quality of life across identified cluster groups were also determined. The R package NbClust was used to determine the number of clusters. It identifies an optimal clustering scheme. It also provides a function to perform k-means and hierarchical clustering with different distance measures and aggregation methods. A combination of validation indices and clustering methods was used by applying a single function which enables the simultaneous evaluation of several clustering schemes while varying the number of clusters, to help determine the most appropriate number of clusters for the data set of interest. Several indices from NbClust were used to compute the number of clusters of BCS problems. These included visualization of the distance matrix, k-means algorithm, hierarchical clustering, NbClust’s clusters, and inspection of the Hubert Index and D index [13, 14]. The optimum number of clusters was determined from the K-means algorithm and hierarchical clustering. Elbow, Silhouette, and gap statistics methods were applied for each of the algorithms. Finally, the number of clusters of problems among survivors from hierarchical clustering was visualized using a tree-based representation of the objects, dendrogram, using the row scores of all scales of the CSPro. The function fviz _dend in R package ggplot2 was used to draw the dendrogram [13–16]. Two clusters from the dendrogram tree were specified by the R function cutree.

Each of the five broad problem categories in the CSPro was compared with the two empirically observed problem clusters, using Bonferroni-corrected t-tests. Means, standard deviations, and statistical differences between the clusters were compared. A Mann-Whitney U-test was used to determine the difference in total FACT_B scores between the two clusters.

## Results

### Sociodemographic and clinical characteristics

The descriptive analysis of demographic variables indicated that the majority of participants were between the ages of 40 and 59, with 23% under the age of 40. The majority were married with a history of 2–3 pregnancies. Three-fourth of survivors completed high school and almost 72% were unemployed. Almost two-thirds of the survivors resided in urban settings. The household income for the majority of survivors (65.4%) was below 5000 RMB. More detailed information can be found in Table 1, which also provides specific information on certain clinical characteristics. As can be seen, more than 90% were diagnosed in the last 5 years. Almost half were diagnosed with stage II breast cancer. The most common treatment was surgery plus chemotherapy (28.6%). Ninety percent of the participants had no family history of cancer. The exact types and doses of treatment were not extracted from the medical record (Table 1).

**Table 1:** Sociodemographic and clinical characteristics (n = 1008)

Characteristics	Subgroup	n	%
Age, y	< 40	232	23.0
	40–49	403	40.0
	50–59	299	29.7
	≥ 60	74	7.3
Marital	Single	82	8.1
	Married	926	91.9
Pregnancy	0	32	3.2
	1	154	15.3
	2	328	32.5
	3	245	24.3
	≥ 4	249	24.7
Education	Primary school	283	28.1
	High school	725	71.9
Employment	Unemployed	725	71.9
	Employed	283	28.1
Occupation type	Unemployed	725	71.9
	Institutional services	151	15.0
	Individual household	27	2.7
	Skilled workers	33	3.3
	Farmer	3	0.3
	Other	69	6.8
Residence	City	443	43.9
	Town	213	21.0
	Rural	352	34.9
Income	< 2000 RMB	272	27.0
	2000–5000 RMB	387	38.4
	5001–10,000 RMB	238	23.6
	> 10,000 RMB	111	11.0
Diagnosis	< 1 year	297	29.5
	1–5 year (s)	649	64.4
	> 5 year (s)	62	6.2
Stage	Stage I	193	19.1
	Stage II	568	56.3
	Stage III	247	24.5
Treatment	Surgery	77	7.6
	Surgery + chemo	288	28.6
	Surgery + radio	14	1.4
	Surgery + radio + chemo	201	19.9
	Endocrine	222	22.0
	Targeted therapy	61	6.1
	Others such as herbal therapy	145	14.4
History of cancer	No	907	90.0
	Yes	101	10.0

Note: 6.49 RMB = $1 USD (2-18-21); occupation type other = those working in private enterprise and soldiers

### Number of clusters

**Fig. 1:**
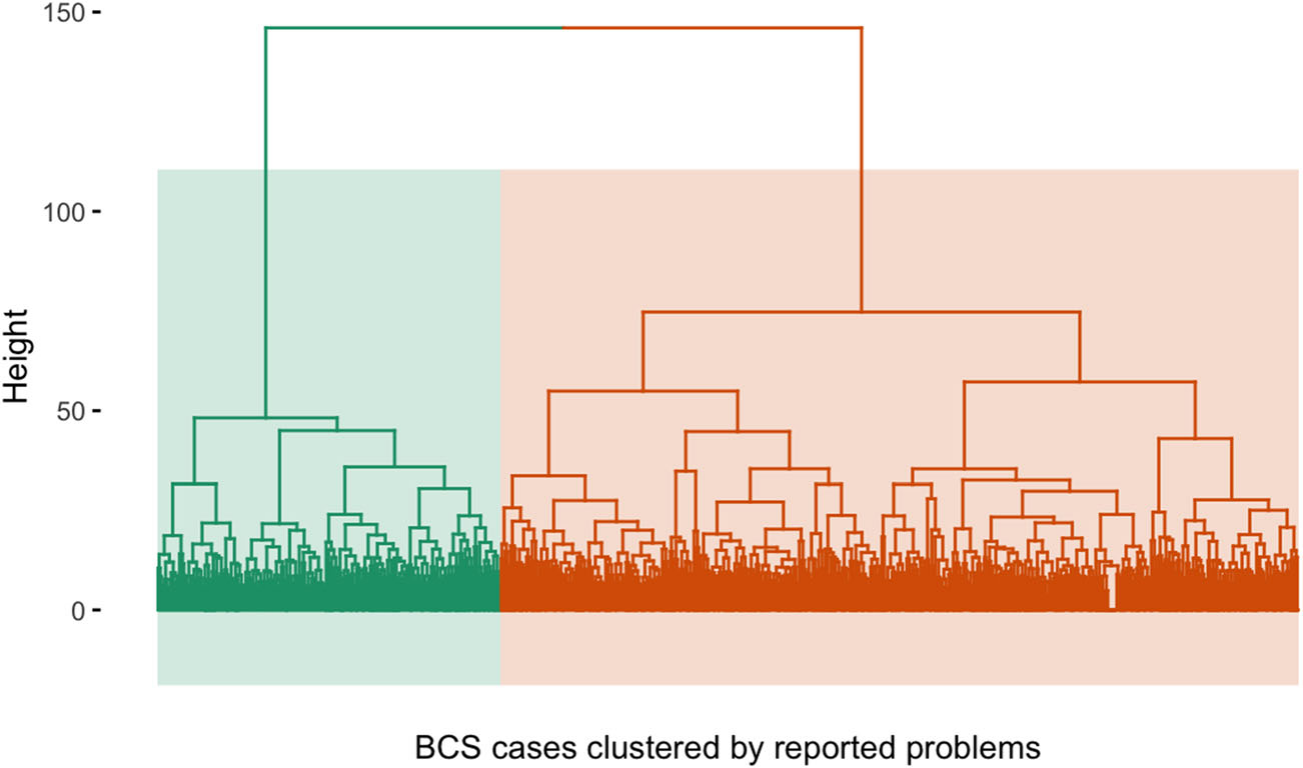
Dendrogram illustrating two clusters. Note. Green-shaded area shows cluster 1 (functional limitation, n = 406) and orange-shaded area displays cluster 2 (multi-problems, n = 602)

The optimum number of clusters was determined from the K-means algorithm and hierarchical clustering. Elbow, Silhouette, gap statistics methods were applied for each of the algorithms. Elbow and Silhouette methods showed that two clusters best represented the BCS, while the gap method generated nine clusters for both the K-means and hierarchical algorithms. The Hubert and D indices were further applied using NbClust algorithm and found that two clusters best represented the multiple problem areas. Akaike and Bayesian information criteria (AIC and BIC) were also determined from the K-means algorithms and Gaussian mixture models, while the Hubert and D indices using the NBClust algorithm found that two clusters best represent the sample. It was also observed that the goodness of fit statistic decreased with each increment in the number of clusters and the rate of decrement was much slower following the two-cluster model. These analyses provided justification for a two-cluster model. Cluster 1 represented 40.3% (n = 406) of the BCS cases while cluster 2 had 59.7% (n = 602) of the cases. The two clusters described the variation in survivorship problems in this relatively large sample of BCS. Table 2 summarizes the goodness-of-fit indices for each cluster model. Figure 1 presents the k-means dendrogram illustrating the two-cluster model.

**Table 2:** Goodness-of-fit indices for cluster models

Model	K-means		Gaussian mixture models	
	AIC	BIC	AIC	BIC
One cluster	71,639.0	71,988.0	203,172.2	203,521.2
Two clusters	59,776.9	60,474.9	189,562.6	190,260.6
Three clusters	56,739.1	57,786.2	185,117.2	186,164.3
Four clusters	54,675.5	56,071.5	182,285.1	183,681.2
Five clusters	53,318.7	55,063.8	180,917.5	182,662.6
Six clusters	52,095.7	54,189.7	177,965.4	180,059.5
Seven clusters	51,031.8	53,475.0	172,937.1	175,380.2
Eight clusters	50,325.5	53,117.7	174,875.2	177,667.3
Nine clusters	49,610.6	52,751.8	167,550.9	170,692.0
Ten clusters	49,180.7	52,670.9	162,322.8	165,812.9

Abbreviations: *AIC*, Akaike’s Information Criterion; *BIC*, Bayes’ Information Criterion

Note: Two clusters were selected according to AIC and BIC and as then indicated above gradually decreased moving from three to ten clusters

### Reported problems across specific domains

Two clusters were significantly different across several domains of problems. As Table 3 illustrates, the problem domains in cluster 1 were best described as those with higher levels of “functional limitations” (problems with cognitive, social, sleep, work, sexual function). Those in cluster 2 were best characterized as BCS with four different problem areas. This cluster was termed “multi-problems” to simply reflect the multiple problems with this cluster. These problems included (1) lower levels of health care–seeking skills (i.e., healthcare competence, patient-provider communication, and health information acquisition), (2) higher level of symptoms (i.e., fear of recurrence, poor body image, pain, fatigue, depressive symptoms, and/or anxiety), (3) greater economic strain, and (4) negative health behaviors (i.e., physical inactivity and unhealthy diet) in contrast to cluster 1 (all p < .001). For a more detailed consideration of the clusters, Table 4 presents each specific problem (subscales) in each cluster and the number of items (questions) to obtain scores for the problems included in the two clusters.

**Table 3:** Differences in problem domains between the two clusters

	Median [IQR]		
Domains	Functional limitations Cluster 1	Multi-problems Cluster 2	p-value
	n = 406	n = 602	
Symptom burden	57.0 [46.3,65.0]	84.0 [78.0, 93.0]	< 0.001
Functional limitations	23.0 [20.0, 28.0]	19.0 [16.0, 23.0]	< 0.001
Health behavior	8.0 [6.0, 9.0]	8.0 [7.0, 10.0]	< 0.001
Economic burden	8.0 [6.8, 12.0]	13.0 [10.0, 16.0]	< 0.001
Health-seeking skills	49.0 [40.0, 55.0]	58.0 [53.0, 66.0]	< 0.001

Abbreviation: *IQR*, interquartile range. The IQR describes the 25th and 75th percentile of values when ordered from lowest to highest

Notes: Because scores were skewed, the median and interquartile range are presented in Table 3 above. The mean and standard deviation stratified by cluster is listed above. Symptom burden: Cluster 1 = 55.78 (12.85), Cluster 2 = 85.98 (12.01); functional limitations: Cluster 1 = 23.46 (5.24), Cluster 2 = 19.39 (5.33); health behavior: Cluster 1 = 7.70 (1.70), Cluster 2 = 8.74 (2.40); economic burden: Cluster 1 = 9.00 (3.85), Cluster 2 = 12.83 (3.67); and health-seeking skills: Cluster 1 = 48.12 (10.23), Cluster 2 = 58.88 (9.70). Higher scores on all measures represent more problems. Mean comparisons, all p’s < 0.001

**Table 4:** Clusters and specific problem areas they represent

Problem areas	Number of items (CSPro)
Cluster I: functional limitations^*^	
(1) Cognitive (Q39–44)	6
(2) Social (Q29–32)	4
(3) Sleep (Q35–38)	4
(4)Work (Q33–34)	4
(5) Sexual (Q45–46)	2
Cluster II: multi-problem areas^#^	
1. Skills to improve quality of health care	
(6) Healthcare competence (Q56–61)	6
(7) Patient-provider communication (Q62–67)	6
(8) Health information (Q69–72)	4
(9) Information acquisition (Q68–73)	6
2. Symptoms	
(10) Fear of recurrence (Q1–6)	6
(11) Body image (Q10–14)	3
(12) Pain (Q10–14)	4
(13) Fatigue (Q15–19)	4
(14) Depressive symptoms (Q21–24)	4
(15) Anxiety (Q25–28)	
3. Health behavior	
(16) Physical inactivity (Q47–48)	2
(17) Healthy diet (Q49–50)	2
4. Financial strain	
(18) Economic burden (Q52–55)	4

Notes: ^*^Ability to function optimally in everyday life’s activity-remember, think, plan, coordinate, interact effectively with others, ability to function at work, ability to sleep and interest in sexual function

^#^There are four major problem areas observed in this cluster. This cluster includes skills helpful for improving the quality of health care. It also includes specific problems in symptom burden, health behaviors, and financial strain.The range represents to exact item numbers of the CSPro

### Cluster-specific sociodemographic and clinical characteristics

Of the 1008 cancer survivors, n = 406 (40.3%) survivors fell into the “functional limitation” cluster while the majority n = 602 (59.7%) fell into the “multi-problem” cluster. The differences in sociodemographic and clinical characteristics by cluster are indicated in Tables 5 and 6. The median age was fairly similar (48 vs 46 years) in both clusters. The “multi-problem” group had a greater number of pregnancies and births than the “functional limitations” cluster. Those more likely to fall into the multi-problem cluster tended to live in the countryside (39.7% vs 27.8%) and had a lower income < 5000RMB (75.8% vs 50%) than the “functional limitations” cluster. Also, the “multi-problem” group tended to be 1–5 years from diagnosis, with stage 2 disease, and exposed to either surgery and chemotherapy or the combination of surgery, radiotherapy, and chemotherapy more often than those with “functional limitations.” The “multi-problem” group was less likely to report a family history of cancer (Tables 5 and 6).

**Table 5:** Socio-economic characteristics per need cluster (n = 1008)

Variable	Group	Functional limitationn = 406		Multi-problems n = 602		p-value
		n	%	n	%	
Age (median, IQR)		48	42–53	46	39–52	< 0.01
Age, y	< 40	77	19.0	155	25.7	0.07
	40–49	165	40.6	238	39.5	
	50–59	132	32.5	167	27.7	
	≥ 60	32	7.9	42	7.0	
Marital	Single	30	7.4	52	8.6	0.553
	Married	376	92.6	650	91.4	
Pregnancy	0	12	3.0	20	3.3	< 0.001
	1	87	21.4	67	11.1	
	2	137	33.7	191	31.7	
	3	81	20.0	164	27.2	
	≥ 4	89	21.9	160	26.6	
No. of children	0	14	3.4	26	4.3	< 0.001
	1	191	47.0	194	32.2	
	2	166	40.9	282	46.8	
	≥ 3	35	8.6	100	16.6	
Education level	Primary	109	26.8	174	28.9	0.521
	High school	297	73.2	428	71.1	
Work	Unemployed	297	73.2	428	71.1	0.521
	Employed	109	26.8	174	28.9	
Occupation	Unemployed	297	73.2	428	71.1	0.010
	Institutional service	66	16.3	85	14.1	
	Individual household	16	3.9	11	1.8	
	Worker	8	2.0	25	4.2	
	Farmer	0	0	3	0.5	
	Other	19	4.7	50	8.3	
Residence	City	177	43.6	266	44.2	< 0.001
	Town	116	28.6	97	16.1	
	Rural	113	27.8	239	39.7	
Income	< 2000RMB	38	9.4	234	38.9	< 0.001
	2000–5000RMB	165	40.6	222	36.9	
	5001–10,000RMB	132	32.5	106	17.6	
	> 10,000RMB	71	17.5	40	6.6	

Note: 6.49 Chinese Yuan or RMB = $1 USD (2-18-21)

Occupation type other = those working in private enterprises and soldiers

**Table 6:** Clinical characteristics per problem cluster(n = 1109)

Characteristics	Group	Functional limitation n = 406		Multi-problems n = 602		p-value
		n	%	n	%	
Diagnosis	< 1 year	81	20.0	216	35.9	< 0.001
	1–5 year	303	74.6	346	57.5	
	> 5 year	22	5.4	40	6.6	
Stage	Stage I	105	25.9	88	14.6	< 0.001
	Stage II	217	53.4	351	58.3	
	Stage III	84	20.7	163	27.1	
Treatment	Surgery	47	11.6	30	5.0	< 0.001
	Surgery and chemo	140	34.5	148	24.6	
	Surgery and radio	8	2.0	6	1.0	
	Surgery + radio + chemo	83	20.4	118	19.6	
	Endocrine	50	12.3	172	28.6	
	Targeted therapy	22	5.4	39	6.5	
	Other	56	13.8	89	14.8	
Family history of cancer	No	351	86.5	556	92.4	< 0.001
	Yes	55	13.5	46	7.6	

### Generic problem groupingtype (cluster) and QoL

The total score of the FACT-B in BCS for those in the “functional limitations” cluster = 141 (95% CI 131–150) was higher than those in the “multi-problem” cluster = 113 (95% CI 104–125). The “multi-problem” cluster reported a significantly poorer quality of life total score than the “functional limitation” cluster, p < .001.

## Discussion

Hierarchical cluster analysis indicated that the broad array of problem areas that can be experienced by BCS fell into two clusters: (1) cases that report higher levels of functional limitations and (2) cases with multiple elevated problem areas, or a pattern characterized by lower levels of health-seeking skills, higher symptom burden, unhealthy lifestyle factors, and financial strain. As expected, the cluster experiencing the greater number of problems also reported a lower quality of life, providing a validation of the clustering of a two problem grouping in BCS. These findings were noted in over one thousand cases with diverse breast cancer pathology, as per medical records, in relatively young Chinese woman diagnosed and treated for breast cancer.

From both a clinical and theoretical perspectives, it is possible to observe clear subgrouping of certain concerns following cancer treatment in BCS. The hierarchical clustering in the current study provides empirical support for this type of subgrouping, indicating that the several problem areas in BCS can fall into two distinct groups or clusters. This approach may assist in the identification of potential underlying mechanisms, or common pathways of these clusters of problems. This framework could help optimize the development and application of interventions in BCS, simplifying how clinicians and researchers go about managing these multi-dimensional problems. For example, it could enable identification of a single target area, likely to have downstream benefits for other related problem areas (vs targeting each individual problem area on its own). While it is unclear just how cluster 1 (“functional limitations”) may exert its influence on some pathways, there are some possibilities that can be hypothesized for cluster 2, based on the problems that did cluster together. For example, when a BCS experiences some or many of the problems within cluster 2, it might be possible to improve levels of health care–seeking skills, which may exert a positive effect on symptoms, lifestyle, and/or financial strain. Such a relationship is only speculative at this time and is in need of direct empirical support.

Symptom science has identified patterns or clusters using reported symptoms as its focus [8]. Reports related to common underlying mechanism(s) in the presentation of symptoms, (i.e., sympathetic nervous system reactivity or immune dysfunction [8]) have been suggestive of treatment options for these symptom clusters. The current study extends this concept to multiple diverse problems beyond symptoms, indicating these diverse problems also fall into clusters. While the identification of such mechanisms was not the goal of the present study, it is intriguing that past research on symptom clusters (e.g., [8]) indicates that attempts to cluster a major problem area are possible. Next steps are to determine the mechanisms underlying the two clusters observed in the current investigation. Modifying such underlying mechanisms of these multidimensional problems might similarly improve the understanding and management of these diverse problems.

In fact, a recent investigation using cluster analysis to identify whether patterns of problems (not symptoms only) post-treatment were observed provides support for the potential of the approach used in the present study [17]. These investigators identified a set of problems in BCS that included lifestyle, self-care, emotional coping, social support, sexual health, complementary services, practical help, fear of recurrence, depression, anxiety, pain, and fatigue. Problem areas were observed and classified into four general clusters. These problem areas were named: cluster 1, “low needs”; cluster 2, “mainly physical needs”; cluster 3, “mainly psychological needs”; and cluster 4, “combined physical and psychological needs.” While this study did not measure the exact problem areas using a priori psychometrically developed scales as in the present investigation, the study did illustrate how hierarchical cluster analysis can be reduced to clusters or combinations of several problem areas (i.e., not simply symptoms). This clustering resulted in the ability to form logical groupings that also suggested either no intervention or the general types of interventions used for each observed cluster. The current study was also successful in reducing multiple problem areas into potentially more manageable clusters. Overall, the findings of both studies indicate that it is possible to conceptually reduce the multiple problems reported by BCS into clusters with two broad dimensions.

The difficulty generalizing these findings to countries other than China and to cancer survivors other than those diagnosed and treated for breast cancer with stages I–III is apparent. Also given the cross-sectional nature of the design, it is not possible to determine the causality between the multi-problem cluster and quality of life. While this study used convenience sampling, the sample was relatively large, randomly recruited from multiple cancer hospital sites, and typical of BCS survivors in China [18]. Given these minor limitations, a theoretically robust finding in which two clusters represent eighteen potential problem areas in BCS was observed.

## Conclusion

While it is possible that other methods or tools to measure problems reported by BCS might generate different clusters than what was observed in the present study, this study did use a range of problem areas that was created from a careful review of problems reported by BCS following treatment. It was this diverse set of problems, identified by precise measures, that were synthesized into two clusters. While the clinical impact of this clustering remains to be determined, the empirical separation of these problems into two clusters suggests that further exploration of these clusters is justified. Future research should determine the common mechanisms underlying each cluster and the potential clinical efficiency and effectiveness of addressing such mechanisms of the multiple problems that are often observed among breast cancer survivors.
